# Effects of Matched Intermittent and Continuous Exercise on Changes of Cardiac Biomarkers in Endurance Runners

**DOI:** 10.3389/fphys.2020.00030

**Published:** 2020-01-31

**Authors:** Feifei Li, Jinlei Nie, Haifeng Zhang, Frank Fu, Longyan Yi, Will Hopkins, Yang Liu, Yifan Lu

**Affiliations:** ^1^College of Physical Education, Hebei Normal University, Shijiazhuang, China; ^2^College of Sports Medicine and Rehabilitation, Beijing Sport University, Beijing, China; ^3^School of Health Sciences and Sports, Macao Polytechnic Institute, Macao, China; ^4^Provincial Key Lab of Measurement and Evaluation in Human Movement and Bio-information, Hebei Normal University, Shijiazhuang, China; ^5^Dr Stephen Hui Research Centre for Physical Recreation and Wellness, Hong Kong Baptist University, Hong Kong, China; ^6^Institute of Sport and Health Sciences, Beijing Sport University, Beijing, China; ^7^College of Sport and Exercise Science, Victoria University, Melbourne, VIC, Australia

**Keywords:** high-intensity intermittent exercise, intensity, biomarkers, cardiac troponin, marathon runners

## Abstract

**Purpose:**

Endurance runners training with high-intensity intermittent exercise might experience damage to cardiac muscle. We have therefore compared changes of cardiac biomarkers after workload-matched intermittent and continuous exercise.

**Methods:**

Twelve endurance runners [11 males, 1 female; means ± SD $\dot{\text{V}}$O_2__max_, 62.4 ± 5.4 ml kg^–1^ min^–1^; velocity of $\dot{\text{V}}$O_2__max_ (v $\dot{\text{V}}$O_2__max_), 17.1 ± 1.4 km h^–1^] completed an intermittent and continuous exercise trial in random order. Intermittent exercise consisted of running at 90% v$\dot{\text{V}}$O_2__max_ for 2 min followed by 50% v$\dot{\text{V}}$O_2__max_ for 2 min, repeated for 92 min. Continuous exercise was performed at 70% v$\dot{\text{V}}$O_2__max_ for 92 min. Blood samples were drawn before and 0, 2, 4, 24, and 48 h after exercise for assay of various cardiac biomarkers. Changes in concentration of biomarkers were averaged for the comparison of intermittent with continuous exercise after adjustment for baseline concentration and exercise intensity expressed as percent of heart-rate reserve (%HRR); magnitudes were assessed by standardization.

**Results:**

There were moderate and large increases in high-sensitivity cardiac troponin-I and -T respectively following exercise. The differences between the increases adjusted to the mean intensity of 78 %HRR were trivial, but at 85 %HRR the increases for cardiac troponin-I and -T were moderately higher for intermittent compared with continuous exercise (factor difference, ×/÷90% confidence limits: 3.4, ×/÷1.9 and 2.1, ×/÷1.8 respectively). Differences in the changes in other cardiac biomarkers were trivial.

**Conclusion:**

Prolonged intermittent exercise is potentially more damaging to cardiac muscle than continuous exercise of the same average running speed at higher average heart rates in endurance runners.

## Introduction

There is increasing evidence that an acute bout of intense exercise can induce a minor and temporary elevation of cardiac-specific biomarkers, including various kinds of cardiac troponin, markers diagnostic of myocardial infarction (Thygesen et al., 2012; Eijsvogels et al., 2016). The elevation of cardiac troponin is affected by exercise intensity (Fu et al., 2009), exercise duration (Eijsvogels et al., 2010), baseline level (Legaz-Arrese et al., 2011), training experience of the participants and age (Tian et al., 2012), gender (Kong et al., 2017), cardiovascular risk factors (Vilela et al., 2014), and environment (Li et al., 2016a). A transient increase in myocardial membrane permeability appears to be responsible, but the mechanism is still under debate (Nie et al., 2010, 2016; Li et al., 2016b). Eijsvogels et al. (2016) concluded that exercise intensity was the strongest single predictor of exercise-induced cardiac troponin elevation, and Richardson et al. (2018) found that cardiac troponin was associated with both mean and peak heart rate during exercise. Gresslien and Agewall (2016) hypothesized that there might be a threshold exercise intensity where cardiac troponin release became more marked.

Athletes experience transiently high intensities of exercise when they perform interval training to enhance performance but the impact of such exercise on the heart in comparison with continuous exercise is unclear (Billat, 2001; George et al., 2012; Tschakert and Hofmann, 2013). Continuous exercise resulted in a greater cardiac troponin concentration than intermittent in sedentary men in a study by Ranjbar et al. (2017) whereas Nie et al. (2018) reported no difference between the effects of intermittent and continuous exercise on cardiac troponin changes in sedentary women. In two studies of endurance athletes, intermittent running produced greater increases in cardiac troponin than continuous running, but the exercise intensity and duration were not carefully manipulated and matched (Stewart et al., 2016; Weippert et al., 2016). Other markers of cardiac muscle damage, including N-terminal pro brain natriuretic peptide (NT-pro-BNP) could also provide information on the relative effects of intermittent and continuous exercise (Scherr et al., 2011). The purpose of this study was therefore to compare the changes of cardiac biomarkers after workload-matched high-intensity intermittent and continuous exercise on experienced endurance runners. It was hypothesized that prolonged intermittent exercise potentially induced more perturbations of cardiac biomarkers in endurance runners.

## Materials and Methods

### Participants

After approval of this study by the local Ethical Committee, 12 (11 males and 1 female) endurance runners [means ± standard deviation (SD): age, 23.5 ± 5.5 years; body mass, 63.3 ± 3.9 kg; height, 170.5 ± 5.5 cm; %body fat, 12.6 ± 3.9%; $\dot{\text{V}}$O_2__max_, 62.4 ± 5.4 ml kg^–1^ min^–1^; velocity of $\dot{\text{V}}$O_2__max_ (v$\dot{\text{V}}$O_2__max_), 17.1 ± 1.4 km h^–1^; peak heart rate, 192 ± 9 min^–1^] were recruited from the Department of Physical Education at a local university. They had no history of disease or cardiac symptoms, none were smokers, none were vegetarians or had any special dietary habits and none had taken any drugs or antioxidant supplements in the month before the study. Training history (5.4 ± 3.4 years), training volume (44 ± 25 km week^–1^), and personal best time in a recent marathon race (186 ± 16 min) were self-reported. An initial medical screening and examination were performed by a team of medical doctors and technicians. All of the participants had normal resting blood pressures and electrocardiographic results. All participants provided their written consent and were fully informed about the purposes, procedures, and potential cardiovascular risks of this study. The study took place in the local sports science research center between October and December in the afternoon. Conditions were similar for each test, with small variations in temperature (20.7 ± 2.3°C), humidity (49 ± 13%). All of the participants refrained from intense exercise and alcohol 48 h before and after each trial and were allowed to ingest pure water freely during tests.

### Study Design

The design was a randomized-order crossover. On their first visit to the laboratory, the runners performed a test for estimation of $\dot{\text{V}}$O_2__max_ and corresponding v$\dot{\text{V}}$O_2__max_. On two subsequent visits, the runners completed either intermittent or continuous exercise. The order in which each participant completed the two trials was selected at random and separated by at least 7 days, during which they trained normally. Blood samples were drawn pre-exercise (−1.5 h), immediately post-exercise (0 h), and at four later time points (2, 4, 24, and 48 h) for measurement of biomarkers.

#### Determination of the $\dot{\text{V}}$O_2__max_ and v$\dot{\text{V}}$O_2__max_

A treadmill (H/p/cosmos Pulsar 4.0, H/p/cosmos Sports and Medical gmbh, Nussdorf-Traunstein, Germany) with a 2% slope was used in the determination of $\dot{\text{V}}$O_2__max_ (Max-1, Physio-Dyne Instrument, NY, United States) and in the exercise trials. After a general 5- to 10-min warm-up with self-set speed, the participants ran at an initial speed of 12 km h^–1^, which was increased by 1 km h^–1^ every 3 min without any pause between stages. When the respiratory exchange ratio reached 1.00, the stages were shortened to 2 min. The test stopped either when the increase in oxygen consumption ($\dot{\text{V}}$O_2_) was less than 2.1 ml kg^–1^ min^–1^ while the respiratory exchange ratio was greater than or equal to 1.15, or when exhaustion was reached. $\dot{\text{V}}$O_2__max_ was recorded as the highest 30-s average value of the recorded $\dot{\text{V}}$O_2_. The corresponding v$\dot{\text{V}}$O_2__max_ was recorded at the minimal speed at which $\dot{\text{V}}$O_2__max_ was reached, as long as this speed was sustained for at least 1 min.

#### Exercise Trials

Each participant completed a 1000-m warm-up at their own pace prior to each trial. In the intermittent trial, each bout consisted of a hard run of 90% v$\dot{\text{V}}$O_2__max_ for 2 min, followed by an easy run of 50% v$\dot{\text{V}}$O_2__max_ for 2 min, 23 bouts and 92 min in total. In the continuous trial, participants ran at 70% v$\dot{\text{V}}$O_2__max_ for 92 min. Running velocities for intermittent trial were 15.4 ± 1.3 and 8.4 ± 0.8 km h^–1^, hard and easy rung respectively; for continuous trial was 12.0 ± 0.9 km h^–1^.

### Measurements

Heart rate (S810, Polar, Finland) were recorded every 2 min during each exercise trial. Rating of perceived exertion on Borg’s scale (Borg, 1982) was recorded at 30, 32, 58, 60, 90, and 92 min during each trial. Venous blood samples of 5 ml were dropped from the antecubital vein by venous punctures, clotted at room temperature and centrifuged at 3000 × *g* for 15 min. The separated serum was then drawn and stored at −80°C for further analysis. These laboratory methods are the standard methods used by the local Hospital Clinical Chemistry Laboratory and have been validated.

The high-sensitivity cardiac troponin-I was analyzed using a commercially available high-sensitive immunochemistry STAT assay from Abbott Diagnostics on an Architect i2000SR (Abbott Diagnostics, Chicago, IL, United States) with the detection limit of 1.6 pg ml^–1^ and 99th percentile of the assay was 26 pg ml^–1^. The high-sensitivity cardiac troponin-T analysis method was based on the new electrochemiluminescence technology and using Elecsys 2010 automated batch analysers (Roche Diagnostics, Mannheim, Germany). The measurement range was 3–1000 pg ml^–1^. The 99th percentile cutoff concentration was 14 pg ml^–1^. NT-pro-BNP was determined by an Elecsys pro-BNP ECLIA on the Modular Analytics E170 analyzer (Roche Diagnostic, Mannheim, Germany) with the analytical range 5–35,000 pg ml^–1^. The corresponding upper reference limit was 125 pg ml^–1^. C-reactive protein (CRP) was measured by immunoturbidometric assay from Sekisui Diagnostics (Tokyo, Japan) on an AU2700 analyzer (Olympus Germany, Beckman Coulter, Krefeld, Germany). The cutoff point was set as 3 mg L^–1^. Serum creatine kinase-MB (CK-MB) and creatine kinase (CK) were detected by DC800 analyzer (Beckman Coulter, Krefeld, Germany) using commercial kit according to electrochemiluminescence technology with cutoff values 25 and 195 U L^–1^, respectively.

### Statistical Analysis

Raw data were presented as means mean ± SD unless otherwise stated. All variables were log-transformed for analysis then back-transformed to express effects as factors, after adjustment for the modifying effects of the pre-exercise concentration and either exercise intensity expressed as percent of heart-rate reserve (%HRR) or training volume, using a spreadsheet (Hopkins, 2017). Changes between the two modes of exercise were compared for the average of post-exercise 0, 1, 4, and 24 h for all biomarkers except for CRP, which was the average of 24 and 48 h. In the absence of thresholds for acute changes in cardiac biomarkers associated with substantial increased risk of morbidity or mortality in endurance athletes, the magnitudes of the changes were assessed using threshold standardized changes of 0.20, 0.60, 1.20, 2.0, and 4.0 for small, moderate, large, very large, and extremely large, respectively (Hopkins et al., 2009; Hopkins, 2019a).

Uncertainty in the estimates of effects are presented as 90% compatibility limits. Probabilistic decisions about true (large-sample) magnitudes accounting for the uncertainty were based on one-sided hypothesis tests of substantial (at least small) effects followed by Bayesian inference. The *p*-value for rejecting an hypothesis of a substantial effect magnitude of a given sign was the area of the sampling distribution of the effect with substantial values of that sign (Lakens et al., 2018), evaluated via log transformation. Effects were considered decisive with a *p*-value threshold of <0.05. If an hypothesis of a substantial magnitude of a given sign was rejected, the *p*-value for the hypothesis of the other sign was interpreted as evidence for that hypothesis, since the *p*-value corresponds to the posterior probability of the magnitude of the true effect in a reference Bayesian analysis with a minimally informative prior (Hopkins and Batterham, 2016; Hopkins, 2019b). This *p*-value is reported qualitatively using the following scale: 0.25–0.75, possibly; 0.75–0.95, likely; 0.95–0.995, very likely; >0.995, most likely (Hopkins et al., 2009). If neither hypothesis was rejected, the magnitude of the effect was considered to be unclear, and the magnitude of the effect is shown without a probabilistic qualifier.

## Results

**FIGURE 1:**
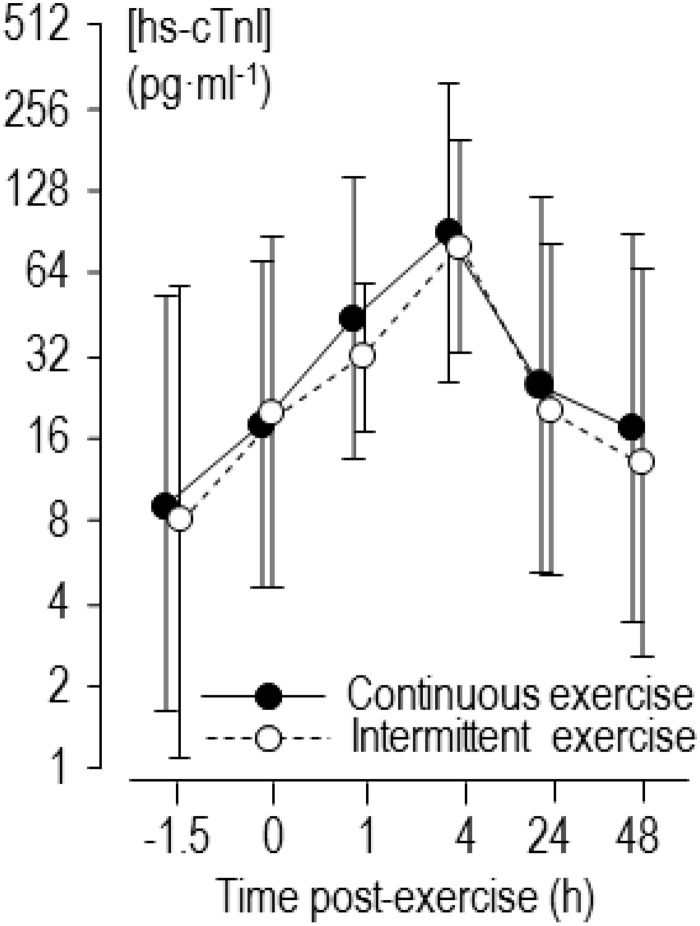
Back-transformed means of log-transformed concentrations of high-sensitivity cardiac troponin I (hs-cTnI) 1.5 h before and 0–48 h after continuous and intermittent exercise. Error bars are standard deviations.

No runners reported any cardiac symptoms during or after the exercises. Heart rate during the intermittent trial was 160 ± 12 min^–1^ (176 ± 12 min^–1^ for the hard running and 145 ± 13 min^–1^ for the easy running); during the continuous trial heart rate was 162 ± 11 min^–1^. The intensities were 78 ± 6 and 79 ± 5 %HRR for intermittent and continuous trials, respectively, or 83 ± 4 and 84 ± 4 %peak heart rate. Rating of perceived exertion during intermittent and continuous trials was 14 ± 3 and 12 ± 3, respectively. Values for pre-exercise concentration of biomarkers are shown in Table 1, and values for high-sensitivity cardiac troponin-I pre- and post-exercise are shown in Figure 1. The baseline level of high-sensitivity cardiac troponin-I in three runners exceeded the upper reference limit of 26 pg ml^–1^, and after intermittent and continuous exercises this limit was exceeded by 11 and 10 runners, respectively.

**TABLE 1:** The pre-exercise concentrations of biomarkers.

	Raw	Back-trans.
	(mean ± SD)	(mean ×/÷ SD)
High-sensitivity cardiac troponin I (hs-cTnI, pg ml^–1^)	70 ± 180	8.6 ×/÷ 6.2
High-sensitivity cardiac troponin T (hs-cTnT, pg ml^–1^)	8 ± 10	5.3 ×/÷ 2.0
N-terminal pro brain natriuretic peptide (NT-pro-BNP, pg ml^–1^)	28 ± 26	18 ×/÷ 2.6
C-reactive protein (CRP, mg L^–1^)	1.1 ± 1.1	0.7 ×/÷ 2.9
Creatine kinase-MB (CK-MB, U L^–1^)	3.9 ± 2.8	3.3 ×/÷ 1.8
Creatine kinase (CK, U L^–1^)	350 ± 250	289 ×/÷ 1.9

*Back-trans.: back-transformed mean and factor SD of the log-transformed concentrations.*

**FIGURE 2:**
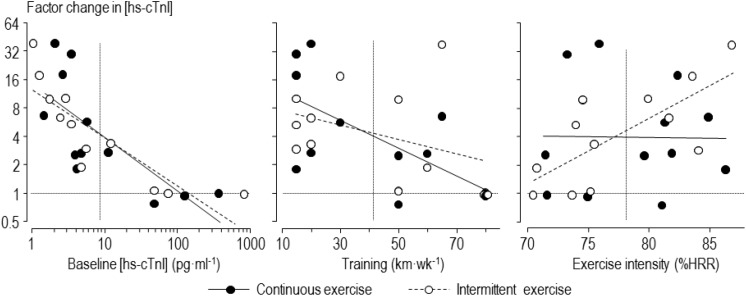
Individual factor changes of high-sensitivity cardiac troponin-I (hs-cTnI) averaged over 0–24 h following intermittent and continuous exercise, with regression lines showing prediction by baseline concentration, training volume and exercise intensity. Horizontal dotted lines indicate no change in concentration; vertical dotted lines indicate mean values of the predictors.

The mean and factor SD for the individual averaged changes in concentrations of biomarkers for the intermittent and continuous trials are presented in Table 2. These changes were large for high-sensitivity cardiac troponin-T, moderate for high-sensitivity cardiac troponin-I and CK-MB, and small for NT-Pro-BNP, CRP, and CK. The individual changes following intermittent and continuous exercise, with regression lines showing prediction by baseline concentration, training volume and exercise intensity, are shown in Figure 2 for one of the biomarkers, high-sensitivity cardiac troponin-I. The figure shows apparently similar modifying effects of baseline concentration and training volume on high-sensitivity cardiac troponin-I, but a marked difference in the modifying effect of exercise intensity.

**TABLE 2:** Changes in concentration of biomarkers following continuous and intermittent exercise, with magnitude-based decisions for the comparison of the changes adjusted to mean values of potential modifiers and to 1 SD above baseline concentration, to approximately 1 SD above mean Tvol (67 km week^–1^), and to approximately 1 SD above mean exercise intensity (85% HRR).

	Factor change scores^a^		Effect for intermittent/continuous^b^			
	(mean ×/÷ SD)		(mean, ×/÷90CL) and magnitude-based decision^c^			
		
	Continuous	Intermittent	At mean values^d^	At baseline + 1 SD^d^	At 67 km week^–1^	At 85 %HRR
hs-cTnI	4.4 ×/÷ 2.4	4.4 ×/÷ 1.8	1.0, ×/÷1.4 trivial, ↔^00^	1.7, ×/÷1.7 small, ↑*	1.8, ×/÷2.0 small,↑*	3.4, ×/÷1.9 moderate,↑***
hs-cTnT	3.0 ×/÷ 2.1	3.2 ×/÷ 1.4	1.0, ×/÷1.4 trivial	1.1, ×/÷1.7 trivial, ↔^0^ ↑*	1.3, ×/÷1.6 trivial, ↔^0^ ↑*	2.1, ×/÷1.8 moderate,↑**
NT-pro-BNP	1.8 ×/÷ 1.3	1.7 ×/÷ 1.2	1.0, ×/÷1.2 trivial, ↔^00^	1.1, ×/÷1.3 trivial, ↔^0^ ↑*	0.9, ×/÷1.4 trivial	1.1, ×/÷1.4 trivial
CRP	1.3 ×/÷ 1.7	1.6 ×/÷ 1.4	1.2, ×/÷1.3 trivial, ↔^0^ ↑*	1.1, ×/÷1.4 trivial, ↔^0^ ↑*	1.1, ×/÷1.3 trivial, ↔^00^	1.3, ×/÷1.4 small, ↑*
CK-MB	1.6 ×/÷ 1.5	1.7 ×/÷ 1.6	1.1, ×/÷1.5 trivial	1.0, ×/÷1.9 trivial	1.0, ×/÷2.0 trivial	1.0, ×/÷1.9 trivial
CK	1.4 ×/÷ 1.4	1.5 ×/÷ 1.5	1.0, ×/÷1.4 trivial	1.2, ×/÷1.6 small, ↑	1.0, ×/÷1.5 trivial	1.1, ×/÷1.7 small, ↑

SD, standard deviation; 90CL, 90% confidence limits as factors; Tvol, training volume, km week^–1^; %HRR, percent of heart-rate reserve; hs-cTnI, high-sensitivity cardiac troponin I, pg ml^–1^; hs-cTnT, high-sensitivity cardiac troponin T, pg ml^–1^; NT-pro-BNP, N-terminal pro brain natriuretic peptide, pg ml^–1^; CRP, C-reactive protein, mg L^–1^; CK-MB, creatine kinase-MB, U L^–1^; CK, creatine kinase, U L^–1^. ^*a*^Changes, adjusted to overall mean values of baseline concentration and intensity, are for the mean of 0, 1, 4, and 24 h post-exercise, except for CRP, which is the change for the mean of 24 and 48 h post-exercise. ^*b*^All effects are adjusted for baseline concentration. ^*c*^The qualitative magnitude shown is derived by standardization for the observed effect. For clear effects, likelihood of a true substantial increase (↑) is indicated as *possible; **likely; ***very likely. Likelihood of a true trivial change (↔) is indicated as follows: ^0^possible; ^00^likely. ^*d*^Means and SD are shown in Table 1. These effects are adjusted for intensity.

Comparisons of the changes in high-sensitivity cardiac troponin-I and in the other biomarkers adjusted to the mean values of the modifiers and to approximately 1 SD above the mean values are shown in Table 2. In summary, there were trivial differences between the two modes of exercise at the mean values of the modifiers, although the differences were not clear for some measures (high-sensitivity cardiac troponin-T, CK-MB, CK). Effects of 1 SD of baseline concentration of the marker and of training volume had at most small observed effects, and true values for the clear effects were at most only possibly substantial. However, intensity approximately 1 SD above the mean of heart-rate reserve (85 %HRR) had clear moderate effects on high-sensitivity cardiac troponin-I and -T, with high likelihoods that the true change with intermittent was greater than that with continuous exercise. With the other biomarkers only CRP showed a clear difference at 85 %HRR (a small, possibly substantial increase with intermittent compared with continuous), effects on the other biomarkers being trivial or small and unclear.

## Discussion

To our knowledge, this is the first study to explore the effect of intermittent exercise on cardiac biomarker concentrations by comparing workload-matched continuous exercise in endurance runners. Our novel findings suggest that there was little difference in the effect of exercise mode on cardiac-troponin elevation at typical training intensity, but for runners exercising at higher relative heart rates, prolonged intermittent exercise is potentially more damaging to cardiac muscle than comparable continuous exercise.

Referring to the mean values of high sensitivity cardiac troponin-I in Figure 1 and Table 1, there was a similar effect of two forms of exercise. Of course, as markers for diagnosis of acute myocardial injury in clinical settings, the actual magnitude of individual cardiac troponin level itself should be considered. The peak level of high-sensitivity cardiac troponin-I exceeded the upper reference limit of 26 pg ml^–1^ in most runners after both forms of exercise. Others have noted that cardiac troponin often exceeds the upper reference limit following exercise (Vilela et al., 2014; Gresslien and Agewall, 2016). Although strongly needed, there are no agreed thresholds for acute changes in cardiac troponin associated with substantial increased risk of morbidity or mortality in athletes, as the clinical relevance of exercise-induced cardiac troponin is still under debate (Skadberg et al., 2017; Nie, 2018). It has been argued previously that cardiac troponin is not randomly elevated after vigorous exercise but increases in certain “susceptible” individuals (Nie et al., 2011; Tian et al., 2014; Legaz-Arrese et al., 2015). We discuss below the extent to which resting concentration of cardiac troponin and level of training are two characteristics that modify release of cardiac troponin following exercise in our study.

Our findings for the effect of intensity of intermittent exercise are consistent with other studies, reviewed in the Introduction, showing greater elevation of cardiac biomarkers with higher intensity exercise. In particular, it obtained pronounced high-sensitivity cardiac troponin-T elevation after intermittent exercise with average heart rate of 160 min^–1^, which was similar to the mean heart rate of our runners (Richardson et al., 2018). Stewart et al. (2016) suggested that a heart rate of 145 min^–1^ was a threshold for the elevation of cardiac troponin. Gresslien and Agewall (2016) and Nie et al. (2018) also presented evidence for a threshold exercise intensity. With the prolonged exercise of trained runners in our study, there is evidence of a threshold heart rate for intermittent exercise, but not for continuous exercise (Figure 2, right-hand scatterplot). The greater increase in cardiac troponin concentrations with intermittent compared with continuous exercise at high relative intensities is obviously due to the higher peak heart rates during the on-phase of each interval; the lower heart rates during the off-phase do not compensate for the on-phase for high relative intensities, but they more than compensate for low relative intensities, on average. Clearly, however, there are individual differences in the response to both types of exercise that are not explained by relative exercise intensity across the range of intensities.

As shown in Figure 2, the baseline level of cardiac troponin and training volume can explain some of the individual differences in response to the intermittent and continuous exercise. The three participants with the highest baseline concentrations of cardiac troponin-I experienced absolutely no increase with either form of exercise, so any cardiac pathology represented by the concentration of this marker is apparently not exacerbated by exercise in these participants. In the analysis that included all participants with adjustment for intensity, there was only a small or trivial mean increase with intermittent compared with continuous exercise at high baseline concentrations of either form of cardiac troponin, and the true increases were only possibly substantial. We therefore do not consider that intermittent exercise is a concern for runners with high baseline values of cardiac troponin, at least with this sample: if anything, baseline concentration had a protective effect for exercise-induced increases. There was a similar protective effect for training volume, again with only a possibly small relative mean increase for intermittent compared with continuous exercise for participants with high training volumes.

We found small increases in NT-pro-BNP after both forms of exercise, with little difference between two exercise modes. NT-pro-BNP is a marker of cardiac stress elicited from volume or pressure overload in clinical settings of myocardial injury (Kociol et al., 2010). The magnitude of increase in NT-pro-BNP is primarily dependent on exercise duration as well as basal concentration, but not exercise intensity (Scharhag et al., 2008; Legaz-Arrese et al., 2011; Serrano-Ostariz et al., 2011). There seems a “ceiling effect” that NT-pro-BNP is maximized at a low exercise intensity so that further increase requires accumulation over time (Legaz-Arrese et al., 2011). We also observed small increases in CRP after intermittent and continuous exercise and possible small effect of exercise mode at 85 %HRR. CRP and other inflammatory markers have been reported to increase 24–74 h after endurance exercise (Scherr et al., 2011). Stewart et al. (2016) also observed CRP increased to a greater extent with intermittent exercise accompanying cardiac troponin elevations. Although this marker is not cardiac-specific, inflammatory cytokines related to cardiac injury have been reported to affect the release of cardiac troponin after exercise (La Gerche et al., 2015). Inflammation may therefore have contributed to the exercise-induced cardiac troponin elevation via a transient increase in myocardial membrane permeability. Further research is needed to address this issue.

## Limitations and Perspective

The unclear comparisons of exercise mode and some of the unclear moderating effects for the markers other than cardiac troponin need resolving with a larger sample size, which would also improve the precision of the estimates of the effect with cardiac troponin. Other factors that may contribute to the individual variation of exercise-induced cardiac troponin elevation have not been verified for the small sample size. Competing at similar intensity, younger age predicts cardiac troponin elevation among endurance runners (Eijsvogels et al., 2015). Sex effect may also influence the individual variation as one female runner was included in the present study. Exercise-induced cardiac troponin elevation occurred in both male and female runners, lower in female (Kong et al., 2017), but not exactly the case in a meta-analysis (Shave et al., 2007). Furthermore, core temperature, diet and fluid intake, which may have impact on the performance of endurance runners were not monitored between the two trial.

Cardiac troponin, a biomarker to diagnosis acute myocardial infarction is moderately to largely increased after typical long hard training in endurance runners. Some runners training with high-intensity intermittent exercise for improved performance may be at increased risk of cardiac stress. Baseline concentration of cardiac biomarkers and training volume have protective effects for cardiac stress of endurance runners participating with high training volumes. Training can be a potentially precondition to better cardiomyocyte tolerance to intense exercise.

## Conclusion

Intermittent and continuous exercise at similar mean heart rates have similar mean effects on elevation of cardiac troponins in marathon runners performing long hard training. Elevation of cardiac troponins is greater at higher intensities and less at lower intensities with intermittent but not continuous exercise. Baseline level of cardiac troponin and training volume can explain some of the individual differences in response to intermittent and continuous exercise.

## Data Availability Statement

All datasets generated for this study are included in the article/supplementary material.

## Ethics Statement

The studies involving human participants were reviewed and approved by the Beijing Sport University (BSUIRB, 2019003H). The patients/participants provided their written informed consent to participate in this study.

## Author Contributions

FL, JN, HZ, FF, LY, and YLu conceived and designed the study. FL, LY, WH, YLi, and YLu performed the experiments and analyzed the data. All authors drafted the manuscript.

## Conflict of Interest

The authors declare that the research was conducted in the absence of any commercial or financial relationships that could be construed as a potential conflict of interest. The handling Editor and reviewer AA declared their involvement as co-editors in the Research Topic, and confirm the absence of any other collaboration.
